# In silico identification of common epitopes from pathogenic mycobacteria

**DOI:** 10.1186/1471-2172-14-S1-S6

**Published:** 2013-02-25

**Authors:** Bárbara de la Caridad Addine Ramírez, Reynel Marrón, Rommel Calero, Mayelin Mirabal, Juan Carlos Ramírez, María E Sarmiento, Mohd Nor Norazmi, Armando Acosta

**Affiliations:** 1 Finlay Institute. Ave. 27, No. 19805, La Lisa. La Habana, Cuba; 2 School of Health Sciences Universiti Sains Malaysia, Malaysia; 3 Institute for Research in Molecular Medicine, Universiti Sains Malaysia, Malaysia

## Abstract

An in silico study was carried out to identify antigens for their possible collective use as vaccine candidates against diseases caused by different classes of pathogenic mycobacteria with significant clinical relevance. The genome sequences of the relevant causative agents were used in order to search for orthologous genes among them. Bioinformatics tools permitted us to identify several conserved sequences with 100% identity with no possibility of cross-reactivity to the normal flora and human proteins. Nine different proteins were characterized using the strain H37Rv as reference and taking into account their functional category, their *in vivo* expression and subcellular location. T and B cell epitopes were identified in the selected sequences. Theoretical prediction of population coverage was calculated for individual epitopes as well as their combinations. Several identical sequences, belonging to six proteins containing T and B cell epitopes which are not present in selected microorganisms of the normal microbial flora or in human proteins were obtained.

## Introduction

There are different species of mycobacteria that cause many human infections with high mortality and morbility [1,2]. Three of the most common diseases caused by them are tuberculosis (TB), leprosy and Buruli ulcer.

Atypical mycobacteria are the causative agents of diseases caused by mycobacteria other than *M. tuberculosis* (MTB) and *M. leprae*. Infections with these species have become more frequent nowadays. HIV infection, socioeconomic conditions and antibiotic resistance are some of the reasons for the increased prevalence of these diseases [3]. This fact may also be associated with the waning protective effect of BCG [4].

The absence of an effective vaccine against mycobacterial diseases [5-7] and its high prevalence in poor countries with low accessibility to health services justified the search for vaccine candidates that can be used to simultaneously protect humans against diseases caused by the different pathogenic mycobacteria [8].

Vaccine design can be expedited via the application of *in silico* techniques combined with immunological methods. Bioinformatics tools enable researchers to move rapidly from genomic sequence to vaccine design. These new tools allow the selection of regions of microbial genomes that are predicted to trigger protective immune responses to be used as components for vaccine constucts [9,10].

The present research aims to identify antigens using *in silico* studies for the development of vaccine candidates that can simultaneously protect humans against diseases caused by pathogenic mycobacteria.

## Materials and methods

The mycobacteria genomes were retrieved from Gene Bank database (http://www.ncbi.nlm.nih.gov/).

A whole–genome alignment was performed using Mauve tools in order to search for orthologous genes with identities ranging from 98 to 100% homology.

A local alignment among all protein sequences for each orthologous genes was perfomed with Clustalw program, and all sequences having identity of more than 20 continuous amino acids were chosen.

Selection of similar genetic regions among mycobacteria and genetic regions from selected bacterial flora of the normal microbiome was also performed.

The amino acid sequences obtained were compared with the available sequences of selected microbiome using the local alignment tool FASTA 36.3.4. The parameters for the program were ktup=2, DNA STRAND=N/A and SEQUENCE= PROTEIN.

Nine agents were selected for the comparison:

Bacteroides fragilis 638R

Bifidobacterium bifidum PRL2010

Clostridium dificile

*Escherichia coli 'BL21-Gold* (*DE3*)*pLysS AG'*

Lactobacillus acidophilus NCFM

Mycobacterium smegmatis str. MC2 155

*Staphylococcus epidermidis ATCC 12228*,

Streptococcus agalactiae 2603V/R

Streptococcus sanguinis SK36.

Finally, sequences below 70% homology and those without theoretical possibilities of forming linear B cell epitopes were selected.

### Prediction of subcellular localization of proteins

The subcelular localization of the selected proteins was defined using the report of the identification and localization of 1044 MTB proteins [11].

Subcelular localization of proteins that did not appear in the report was predicted using three servers: PSORTb [12] , TBpred, [13] and SignalP [14].

### Identification and selection of *in-vivo* expressed genes

A bibliographic search was performed to look for reports of *in-vivo* studies of MTB gene expression in humans and animals using google scholar, PubMed and PubMed Central.

### HLAPred server was used for prediction of T-cell epitopes in this study

Thirty six HLA class I and 51 HLA class II were selected for the prediction. The results were displayed in HTML Mapping form and the threshold of 3% were use as default for the prediction parameters [11]. T cell epitopes similar to human epitopes were eliminated.

### B-cell epitope prediction

Two servers, Bcepred and ABCpred, were combined for the prediction of linear B cell epitomes. For Bcepred server: Seven physico-chemical properties of amino acids (hydrophilicity, flexibility, accessibility, polarity, exposed surface and turns and antigenic propensity) were combined with a threshold at 2.38. For ABCpred server: A threshold of 0.5 and the predicted B cell epitope length of 16 amino acids were used [12]. The regions containing both T and B cell epitopes were selected by BioHelper tools [15].

The epitopes analyzed were compared with the report of Iñakis et al. [16] for eliminating hyperconserved epitopes.

### Population coverage calculation of individual epitopes and their combinations

The theoretical prediction of the presentation of the individual epitopes and their combinations for MHC alleles of Cuban, Malaysian, Brazilian, Mexican, Australian, African, and North American populations were calculated using the Population Coverage Calculation program [18].

**Figure 1:**
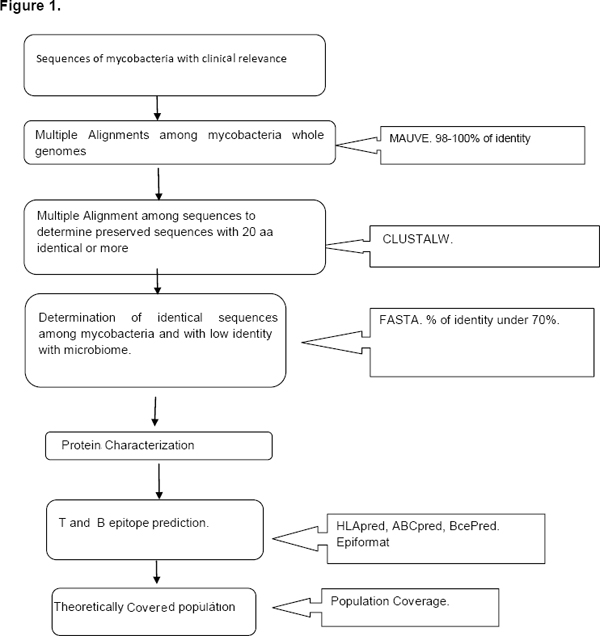
Flowchart of the procedure –bioinformatics analysis

The percentage of coverage greater than 70% was accepted as good consistent with reports in the literature [18,10,19,11,14] (figure 1).

## Results

Thirteen-whole mycobacteria genome alignments were performed and 26 orthologous genes were determined. A local alignment among each protein sequence for each orthologous gene was performed and 71 sequences were obtained with more than 20 identical amino acids between them, corresponding to 14 proteins.

In order to avoid the selection of epitopes share with the host microbiome only, only 20 epitopes were retained in this study. It was found that 13 of 14 proteins were expressed in the membrane; with six only expressed in the membrane, two in the membrane and cell wall; three in the membrane and cytosol and two in all of these compartments. One protein was only expressed in the cytosol [20,21].

Table 1 shows a characterization of predicted proteins containing sequences showing 100% identity among pathogenic mycobacteria, and not shared with human proteins or the normal human microbiome. These proteins have also been predicted to contain T and/ or B cell epitopes, and are expressed during infection.

**Table 1:** Characterization of predicted proteins

Identification (ORF)	T epitopes	B epitopes	Up-regulation during infection
Rv0667	YES	YES	YES

Rv1299	YES	NO	YES

Rv1315	YES	YES	NO

Rv1420	YES	NO	NO

Rv1421	YES	YES	NO

Rv1547	YES	YES	YES

Rv0701	NO	YES	NO

Rv1308	NO	YES	NO

Rv1384	NO	YES	YES

Four of the identified genes (Rv1269, Rv0667, Rv1547 and Rv1384) have been reported to be up-regulated during infection. Rv1299 is over-expressed during infection in human macrophages, mice and guinea pig lungs [22]. Rv0667, Rv1547and Rv1384 are over-expressed only during infection in guinea pig lungs [23].

The selected sequences from Rv0701, Rv1384 and Rv1308 are not predicted to contain T cell epitopes but were chosen based on reports of their potential role in the humoral immune response against MTB [23-25].

Hyperconserved epitopes are not present in the selected sequences. These sequences were excluded to avoid regions, which may be selected during the co-evolution of MTB and humans and hence may provide an advantage to the pathogen [17].

Five sequences that contain epitopes similar to human epitopes were eliminated [13] to avoid potential autoimmune response.

In this research, the identified sequences belonging to 15 MTB proteins and some of its combinations were analyzed. The combination of all sequences were predicted to give more than 70% population coverage when presented via the MHC class I plus MHC class II molecules in the selected geographical regions. In contrast, the predicted population coverage of individual sequences was highly variable in the different geographical regions and was always bellow 70 % (data not shown). However, these sequences were not excluded because of their potential use in the specific geographical regions.

**Figure 2:**
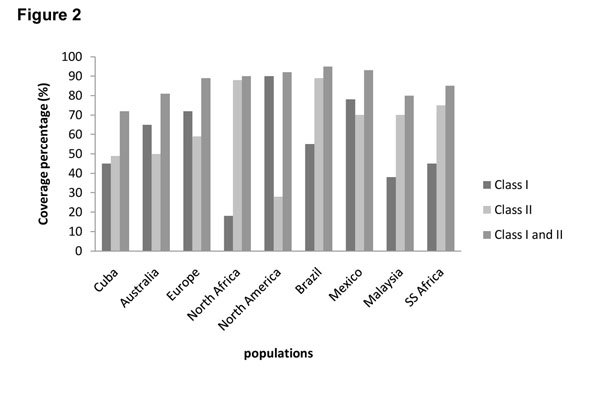
Population coverage for combination of all sequences

The combination of all sequences shows population coverage over 70 % in all geographical regions studied (Figure 2).

Evaluation of the identified sequences in *in vitro* and in animal models should be carried out to verify their utility to be included in vaccine formulations.

## Conclusions

We used bioinformatics tools to predict sequences, which are common among different mycobacteria belonging to the MTB complex and are absent from selected microorganisms of the normal flora. The predicted sequences comprise those that are expressed primarily in the membrane and contain B and T cell epitopes that do not match with the human epitopic database. The combination of the identified sequences gives appropriated theoretical population coverage in the different geographic regions studied. These predicted sequences are potential vaccine candidates but functional/biological assays should be performed to verify whether they are indeed appropriate to be included in a vaccine formulation.

## Competing interests

The authors declare that they have no competing financial interests.

## Authors' contributions

All authors have read and approved the final manuscript BCAR, performed the bioinformatics studies and participated in data analysis and in writing of the manuscript. RC developed softwares used in the bioinformatics studies, performed the bioinformatics studies and participated in data analysis and in writing of the manuscript. RM,MM, JCR helped in the bioinformatics studies. MES, MNN, AA conceived the study, participated in data analysis and in writing of the manuscript.

## Declarations

This article has been published as part of *BMC Immunology* Volume 14 Supplement 1, 2013: Proceedings from Delivery Systems and Current strategies to drug design. The full contents of the supplement are available online at http://www.biomedcentral.com/bmcimmunol/supplements/14/S1
